# Fabrication of Alginate/Chitosan Composite Beads for Improved Stability and Delivery of a Bioactive Hydrolysate From Shrimp ( *Litopenaeus vannamei* ) Head

**DOI:** 10.1002/fsn3.70443

**Published:** 2025-06-17

**Authors:** Sampurna Rai, Kanyawee Whanmek, Ploypailin Akanitkul, Angkansiri Deeaum, Thunnalin Winuprasith, Varongsiri Kemsawasd, Uthaiwan Suttisansanee, Chalat Santivarangkna, Suwapat Kittibunchakul

**Affiliations:** ^1^ Master of Science Program in Food Science for Nutrition (International Program), Institute of Nutrition Mahidol University Nakhon Pathom Thailand; ^2^ Institute of Nutrition Mahidol University Nakhon Pathom Thailand

**Keywords:** encapsulation, food application, functional ingredients, health, *Litopenaeus vannamei*, waste management

## Abstract

This study aimed to encapsulate shrimp head protein hydrolysate (SPH) derived from *Litopenaeus vannamei* using composite alginate/chitosan hydrogels for potential food applications. SPH‐loaded microparticles were produced via ionic gelation of 3% (w/v) alginate and 0.25% (w/v) chitosan in the presence of calcium lactate, achieving the highest encapsulation efficiency of 61% and an average diameter of ~1.5 mm. These beads exhibited pH‐responsive behavior during in vitro digestion. They could withstand gastric conditions and showed a burst protein release of > 72% upon transition to the intestinal phase, resulting in ~95% recovery of SPH in simulated small intestinal fluid. Air‐drying at 50°C for 18 h preserved both the structural integrity of the beads and the bioactivities of encapsulated SPH, making it a viable strategy for prolonging the beads' shelf‐life. Air‐dried beads (~4% moisture content) possessed swelling capacity and stability at both acidic and alkali pH levels, but disintegrated rapidly at pH 7.0. Antioxidant, anti‐hypertensive, and anti‐obesity activities of the encapsulated SPH were significantly better maintained over 4 weeks of refrigeration compared to its non‐encapsulated counterpart, highlighting the protective role of the alginate/chitosan matrix during storage of the beads. These results support the valorization of shrimp‐processing wastes as a source of functional ingredients for incorporation into food and pharmaceutical products. Future research should focus on characterizing the bioactive peptide profiles of SPH and elucidating their interactions within encapsulation matrices. In vivo studies are also needed to validate the current findings and inform the rational design of targeted delivery systems for specific food‐related applications.

## Graphical abstract

Shrimp head protein hydrolysate (SPH) was encapsulated in a composite alginate/chitosan polymer. The composite beads served as a suitable carrier for targeted protein delivery to the intestine. The beads containing SPH possessed high potential for incorporation into acidic food products.

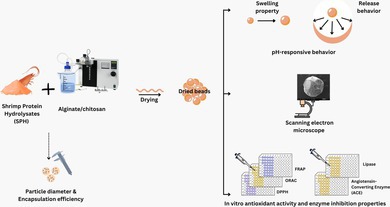

## 1 Introduction

The rapid expansion of the shrimp industry has led to a substantial increase in processing wastes, with shrimp heads, shells, and tails collectively contributing several million tons annually. These wastes are often discarded, posing serious environmental concerns and representing a loss of potentially valuable resources (Kemsawasd et al. 2024). Shrimp‐processing wastes are rich in diverse biomaterials, including chitin, nutrients, carotenoids, and flavor components (Mao et al. 2017). While shrimp shells currently serve as a chitinous substrate for biopolymer production, other components, particularly shrimp heads, remain largely underutilized despite their favorable biochemical composition. Shrimp heads constitute a significant portion of the total processing waste and are notably rich in high‐quality proteins, containing all nine essential amino acids. Proteins account for approximately 50%–65% of the shrimp head's dry weight (Cahú et al. 2012). These proteins can be recovered as soluble hydrolysates through hydrolysis processes, thereby unlocking their nutritional and functional potential for a wide range of applications (Mao et al. 2017). Moreover, the production of protein hydrolysates from industrial waste substrates is regarded as an effective waste management strategy that promotes resource efficiency and minimizes environmental impact (Mahmoud et al. 2007). This dual benefit has driven increasing interest from both industry and academia in developing innovative approaches to convert shrimp heads and their derived protein hydrolysates into functional ingredients.

Biological treatment of shrimp‐processing wastes through enzymatic hydrolysis and/or microbial fermentation enables the production of protein hydrolysates enriched with bioactive peptides that improve health functions and prevent the development of chronic diseases (Ghorbel‐Bellaaj et al. 2018). Protein hydrolysates derived from shrimp heads exhibit multiple beneficial effects, such as antioxidant and antibacterial activities (Nguyen et al. 2024; Zhou et al. 2023), as well as inhibitory activities against enzymes related to some non‐communicable disorders, including angiotensin‐converting enzyme (ACE) for hypertension (Zhou et al. 2023), α‐amylase for type‐2 diabetes (Nguyen et al. 2024), and pancreatic lipase for obesity (Kemsawasd et al. 2024). Despite their potential health benefits, protein hydrolysates, typically produced in aqueous media, are perishable due to their high moisture content. Additionally, the hydrolysis of peptide bonds increases their hygroscopicity by exposing polar functional groups that readily bind water molecules (Ma et al. 2014). As a result, protein hydrolysates often retain physicochemical instability even after drying. Furthermore, it is well recognized that many bioactive peptide constituents within protein hydrolysates are susceptible to gastrointestinal degradation prior to absorption through the intestinal membrane, which may significantly reduce their physiological effectiveness (Aguilar‐Toalá et al. 2022). These limitations can hinder the direct incorporation of protein hydrolysates into foods and beverages, thereby underscoring the need for protective carrier systems capable of enhancing their stability, modulating their release, and preserving their functional properties.

Encapsulation offers a competitive strategy to address the challenges of incorporating protein hydrolysates and peptides into food products by enhancing their stability during storage and improving their bioavailability through protection against gastrointestinal degradation and undesirable interactions with food components (Aguilar‐Toalá et al. 2022). Among encapsulation techniques, ionotropic gelation involving the formation of hydrogel beads through cross‐linking interactions between an ionic polymer and an oppositely charged ion is well‐known for its simplicity and low operating costs (Gadziński et al. 2023). Natural polysaccharides are ideal polymeric carriers for the entrapment of bioactive peptides because they are non‐toxic, structurally stable, and inexpensive, with alginate being one of the most promising choices for encapsulation by ionotropic gelation (Alvarado et al. 2019; Mohan et al. 2015; Sáez et al. 2015). Ma et al. (2014) reported the effective use of an alginate‐based carrier system to reduce the hygroscopicity of whey protein hydrolysate without impairing its immunoregulatory activity, while Kanbargi et al. (2017) demonstrated that encapsulation of protein hydrolysate from lima bean in alginate beads resulted in significant improvements in its antioxidant potential and storage stability. Recent reports suggested that alginate‐based hydrogels provided effective protection for bioactive peptides and allowed their controlled release in the gastrointestinal tract (Alvarado et al. 2019; Thongcumsuk et al. 2023). The rigidity of alginate beads can be improved by incorporating cationic polymers like chitosan, thus enhancing their stability during processing and gastrointestinal transit (Sáez et al. 2015).

Currently, scant information is available on the encapsulation of biologically active compounds obtained from shrimps and their processing wastes. Existing evidence reveals the effective use of encapsulation techniques to stabilize astaxanthin‐rich shrimp extracts (Gómez‐Estaca et al. 2018; Montero et al. 2016). The encapsulation of shrimp waste‐derived protein hydrolysates, as well as their stability and release characteristics, has not been previously explored. Given that shrimp head protein hydrolysates (SPHs) are rich in bioactive peptides with health‐promoting properties, they represent a promising yet underutilized functional ingredient (Kemsawasd et al. 2024). However, their broader application is limited by poor physicochemical stability and low bioavailability. Thus, this study encapsulated a SPH derived from Pacific white shrimp ( *Litopenaeus vannamei* ) by ionotropic gelation of an alginate‐chitosan hybrid hydrogel, and the release behavior, swelling property, and surface morphology of the resulting alginate/chitosan composite beads were investigated. In vitro bioactivities (anti‐oxidation, anti‐hypertension, and anti‐obesity) and the storage stability of free and encapsulated SPH samples were compared. Knowledge gained from this study will pave the way for industries to utilize SPHs by incorporating them into food and pharmaceutical products.

## 2 Materials and Methods

### 2.1 Materials and Chemicals

Analytical‐grade sodium alginate and calcium lactate were obtained from Sigma‐Aldrich (St. Louis, MO, USA). Food‐grade chitosan (molecular weight ~ 800–1000 kDa) was supplied by Asianbioplex AP Operations (Chonburi, Thailand). All chemicals used in the investigations of swelling property, release behavior, and in vitro bioactivities were purchased from Sigma‐Aldrich and were of the highest quality available unless otherwise specified. Deionized water was used to prepare all the solutions. The bioactive SPH used in this study was obtained by fermenting Pacific white shrimp head using food‐grade bacterial cultures (Kemsawasd et al. 2024), as described in Supporting Information. The SPH was lyophilized and stored in vacuum‐sealed foil bags at −20°C. Before use, the SPH was reconstituted with deionized water to a concentration of 1 mg of protein per mL. The proximate composition and zeta potential of the reconstituted SPH are presented in Table S1 and Figure S1, respectively.

### 2.2 Preparation of Alginate/Chitosan Composite Beads

Hydrogel beads were fabricated using an ionotropic gelation technique, following the method of Zhu et al. (2023) with some modifications. Alginate carrier systems (2.0% and 3.0% w/v) were prepared in reconstituted SPH and stirred for 1 h to obtain homogeneous mixtures. Chitosan solutions (0.25%, 0.50%, and 0.75% w/v) were prepared in 5% (v/v) acetic acid containing 7.5% (w/v) calcium lactate and adjusted to pH 5.5. These bead formation conditions were determined based on our preliminary experiments. Spherical beads were generated by dripping the mixture solution of alginate and SPH into the chitosan solution at ambient temperature using an encapsulator (Model B‐390, BÜSHI, Flawil, Switzerland). The device was operated with a 450 μm vibrating nozzle, a vibration frequency of 1000 Hz, an electrode potential of 1500 V, and a pressure of 166 mbar. The beads were allowed to gel for 30 min with continuous mild stirring and subsequently incubated in fresh alginate solution (2.0% or 3.0% w/v) for 20 min to promote cross‐linking (Takka and Gürel 2010). The beads were then harvested and rinsed with deionized water 3 times. A certain amount of the beads was analyzed promptly, and the remainder was air‐dried in an incubator (Model IN110, Memmert GmbH, Schwabach, Germany) at 50°C for 18 h and then stored in a closed, dry container at 4°C. The moisture contents of freshly prepared and dried beads were determined using a moisture analyzer (Model HE53, Mettler Toledo, Kowloon, Hong Kong).

### 2.3 Determination of Bead Diameter and Encapsulation Efficiency

The particle diameter of freshly prepared beads containing SPH was measured (*n* = 100) using a digital Vernier caliper (minimal distance = 0.01 mm) (Narin et al. 2020). The encapsulation efficiency (EE), which refers to the proportion of SPH successfully entrapped within the beads relative to the total amount initially used in the encapsulation system, was determined by dissolving 1 g of the beads in 100 mL of 0.1 M phosphate buffer solution (PBS; pH 7.4) with continuous stirring at 4°C until complete dissolution. The protein content in the resulting solution was analyzed using a 96‐well microplate reader (Model Synergy HT, BioTek Instruments, Winooski, VT, USA) at 660 nm by the Lowry method (Lowry et al. 1951). Empty beads without SPH were also determined by the Lowry method to eliminate the interference of the carrier material. The percentage EE of the beads was calculated using the following equation (Peng et al. 2016), and a hydrogel formulation rendering the highest EE was chosen for fabricating the so‐called SPH‐loaded beads used in further experiments.

$$\%\mathrm{EE}=\frac{\text{Protein in the solution of dissolved beads}}{\text{Total protein added to the encapsulation system}}\times 100$$

### 2.4 Investigation of Release Behavior

The release of encapsulated SPH from the alginate/chitosan composite beads under gastric and intestinal conditions was investigated in vitro using the INFOGEST static gastrointestinal model (Brodkorb et al. 2019). In brief, 3 g of the SPH‐loaded beads were mixed with 50 mL of simulated gastric fluid (SGF) containing pepsin from porcine gastric mucosa (2000 U/mL) and CaCl_2_ (150 μM). The mixture was adjusted to pH 3.0 using HCl and incubated in a shaking incubator (Model ZWYR‐2102C, Labwit Scientific, Victoria, Australia) at 37°C, 130 rpm for 120 min. Then, NaOH solution was added to adjust the pH of the mixture to pH 7.0. This inactivated the pepsin and also simulated the pH of the small intestine. The resulting gastric chime was then incubated with an equal volume of simulated intestinal fluids (SIF) containing bovine bile salt (10 μM) and porcine pancreatin (100 U_trypsin_/mL) at 37°C, 130 rpm for 120 min. The pH was maintained at 7.0 by titrating NaOH solution into the reaction vessel (Rungraung et al. 2022). At defined time intervals, the soluble digested content was aliquoted and centrifuged (10,000 × *g*, 5 min) for further protein determination by the above‐mentioned Lowry method, using the simulated digestive fluid of empty beads as a control. The in vitro release kinetics of the encapsulated SPH in simulated gastrointestinal environments were reported as the percentage of cumulative protein release.

### 2.5 Investigation of Swelling Property

The SPH‐loaded beads were subjected to air‐drying (50°C, 18 h), and the swelling property of the dried beads was investigated at different pH levels, following Zhang et al. (2016) with some modifications. Dried SPH‐loaded beads (0.2 g) were immersed in 200 mL of 10 mM pH‐adjusted PBS (pH 1.0, 3.0, 7.0, and 9.0) at room temperature. At predetermined intervals (0–6 h), the beads were collected, blotted gently with paper towels, and then weighed. The swelling ratio (*Q*s) of the beads was calculated as $Q_{s}=\frac{W_{s}-W_{d}}{W_{d}}$, where *W*_*s*_ is the weight of rehydrated beads at a particular time interval, and *W*_d_ is the weight of dried beads following Lima et al. (2018). The migration of protein through the polymeric matrix of the beads during the swelling period was also investigated by subtracting the protein liberated in PBS from the total protein loaded. The results were expressed as the relative percentage of protein content: $\frac{C_{t}}{C_{i}}\times 100$, where *C*_i_ is the initial protein content encapsulated in the beads, and *C*_*t*_ is the protein content remaining within the beads at different time points.

### 2.6 Surface Morphology Analysis

The surface morphology of fresh, dried, and rehydrated (in PBS at pH 3.0 for 2 h) SPH‐loaded beads was observed under a scanning electron microscope (Model Quanta 250, FEI Company, Hillsboro, OR, USA). The beads were mounted on an aluminum stub and coated with platinum to make them electrically conductive. The morphology of the beads was viewed at an accelerating voltage of 20 kV using a vacuum model (Mohamed et al. 2017).

### 2.7 Investigation of Bioactivities

The antioxidant, anti‐hypertensive, and anti‐obesity activities of free and encapsulated SPHs were investigated in vitro. To measure the activities of encapsulated SPH, a certain amount of fresh or dried SPH‐loaded beads was dissolved completely in 0.1 M PBS (pH 7.4) with continuous stirring at 4°C as aforementioned. The solution was centrifuged (10,000 × *g*, 4°C, 5 min) and promptly analyzed. The biochemical stability of SPH during storage was also explored by refrigerating free SPH and dried SPH‐loaded beads in transparent plastic containers at 4°C for 4 weeks. During this time, samples were taken for periodic determination of the antioxidant, anti‐hypertensive, and anti‐obesity activities. The bioactivity and storage stability assays of empty beads were also performed, and the results were subtracted from the activities of SPH‐loaded beads.

#### 2.7.1 Antioxidant Activity

The antioxidant activities were determined using the oxygen radical absorbance capacity (ORAC), ferric ion reducing antioxidant power (FRAP), and 2,2‐diphenyl‐1‐picrylhydrazyl (DPPH) radical scavenging assays. These assays followed previously described protocols by Sripum et al. (2017) with no modifications. Briefly, the ORAC assay was performed through the reaction between SPH and AAPH radical solution at 37°C. The antioxidant potential of the SPH was observed as the kinetics of fluorescein decay at excitation and emission wavelengths of 485 and 528 nm, respectively. The FRAP assay was conducted by incubating an SPH sample with FRAP reagent at 25°C for 8 min. As an end‐point assay, the reduction of ferric ion to ferrous ion was detected spectrophotometrically at 600 nm. The DPPH radical scavenging assay was conducted by incubating an SPH sample with DPPH radical solution at 25°C for 30 min. The reduction of unstable DPPH molecules relating to antioxidant activity was measured spectrophotometrically as an end‐point assay at 520 nm. Results obtained from all the antioxidant activity assays were reported as μmol Trolox equivalent (TE) per g of protein.

#### 2.7.2 Enzyme Inhibitory Activities

Anti‐hypertensive and anti‐obesity effects were studied in vitro through the inhibition of the key enzymes that control the diseases. Enzyme inhibitory activities were calculated as percentages of inhibition at a particular protein concentration as follows:

$$\%\text{Inhibition}=\left(\frac{B-b}{A-a}\right)\times 100$$

where *A* is the initial rate of the control reaction with the enzyme but without SPH, *a* is the initial rate of the control reaction without the enzyme and SPH, *B* is the initial rate of the enzymatic reaction with SPH, and *b* is the initial rate of the reaction with SPH but without the enzyme.

The inhibitory activity against ACE, the enzyme associated with hypertension, was investigated following the protocol described by Chupeerach et al. (2021). The enzyme reaction comprised 50 μL of SPH sample, 3 μL of ACE from rabbit lung (0.5 U/mL, ≥ 2 U/mg), and 30 μL of 3 mM hippuryl‐histidyl‐leucine in 50 mМ potassium phosphate buffer (KPB; pH 7.0) containing 0.025 NaOH and 3 M NaCl. The mixture was incubated in the dark at 37°C for 30 min and subsequently mixed with 177 μL of 0.28 M NaOH. Then, 15 μL of *o*‐phthaldialdehyde solution (20 mg/mL) and 25 μL of 3 M HCl were sequentially added to visualize and neutralize the reaction, respectively. Reaction results were read on a microplate reader as an end‐point assay at excitation and emission wavelengths of 360 nm and 485 nm, respectively.

The inhibitory activity against lipase, the enzyme associated with obesity, was investigated following the report of Sirichai et al. (2022). The enzyme reaction consisted of 40 μL of SPH sample, 100 μL of lipase type VII from *Candida rugosa* (5 μg/mL, ≥ 700 U/mg), 10 μL of 16 mM 5,5′‐dithiobis(2‐nitrobenzoic acid), and 50 μL of 0.2 mM 5,5′‐dithiobis(2‐nitrobenzoic‐N‐phenacyl‐4,5‐dimethyyhiazolium bromide). The reaction was monitored on a microplate reader as a decline in enzyme kinetics at 412 nm.

### 2.8 Statistical Analysis

Data were expressed as mean ± standard deviation (SD) of triplicate experiments. When needed, one‐way analysis of variance (ANOVA) and Duncan's multiple range test were employed to detect differences between the mean values. An unpaired *t*‐test was used to compare significant differences between the two data sets. Values were considered significantly different at *p* < 0.05. The statistical analyses were conducted using SPSS software Version 23.0 (SPSS Inc., Chicago, IL, USA).

## 3 Results and Discussion

### 3.1 Particle Diameter and EE

The average particle diameter and EE of hydrogel beads obtained using different concentrations of alginate and chitosan are shown in Table 1. The spherical SPH‐containing particles formed using alginate concentrations of 2.0% and 3.0% (w/v) were similar in size (diameter ~ 1.1 mm) and exhibited low EE at 15.28% and 27.17%, respectively. The incorporation of chitosan during the gelation process resulted in significant increases in particle diameter (1.2–1.5‐fold) and EE (1.8–3.0‐fold) compared with gelation using alginate alone. The increased diameter observed with improving EE was attributed to electrostatic interactions between the carboxylic groups of alginate and the amino groups of chitosan, which enhanced the structural integrity of the composite beads and allowed greater entrapment (Umaredkar et al. 2018). The improvement in EE was associated with the impregnation of SPH in the alginate‐chitosan complex, whereby the anionic SPH formed a coacervate with the cationic chitosan (Mohan et al. 2015). Particle diameter and EE were not influenced by increasing chitosan concentrations from 0.25% w/v to 0.75% w/v. This finding concurred with Zhu et al. (2023), who demonstrated that the binding of chitosan to the calcium alginate network was saturated when the chitosan concentration reached 0.30% w/v due to competition between the chitosan and calcium ions for available sites on the alginate chain. In the presence of chitosan, beads containing SPH obtained with 3% (w/v) alginate possessed superior diameters and EE compared with beads obtained with 2% (w/v) alginate (up to 1.2‐fold and 1.4‐fold, respectively), concurring with the results from previous studies (Thongcumsuk et al. 2023; Zhu et al. 2023). Increasing the alginate concentration enabled a higher degree of intermolecular interactions with chitosan and created a tight, low‐porous surface layer within the composite beads, thereby reducing the loss of active core material during the gelation process and potentially enhancing the EE (Ren et al. 2017). Among the alginate composites tested, the highest EE of 61% (~0.61 mg of protein per g of beads) with an average bead diameter of 1.53 mm was observed in beads prepared by dropping 3% (w/v) alginate into calcium lactate solution containing 0.25% (w/v) chitosan. This alginate/chitosan composite was selected for further production and characterization of the SPH‐loaded beads. The particle size of the SPH‐loaded beads categorized them as microcapsules (diameter < 2000 μm), with the feasibility of incorporating the beads into food products without negative impacts on the sensory attributes (Calderón‐Oliver and Ponce‐Alquicira 2022). The maximal EE of the SPH‐loaded beads fabricated in this study was higher than reported for alginate beads loaded with fish‐derived peptides (~54% EE, ~2.0–3.0 mm bead diameter) (Gallegos‐Tintoré et al. 2024) and alginate/chitosan composite beads carrying superoxide dismutase (~52% EE, ~1.7 mm bead diameter) (Zhu et al. 2023). However, our EE was lower than the results obtained from the encapsulation of spirulina protein hydrolysate within the same carrier material (~86% EE, ~1.5 mm bead diameter) (Thongcumsuk et al. 2023). The formulation of alginate/chitosan composite beads is dependent on several factors, such as the properties of the core material, gelation conditions (temperature, pH, mechanical stress), the concentrations of sodium alginate and chitosan, calcium solution, and the contact time of the beads with the solution (Thongcumsuk et al. 2023; Umaredkar et al. 2018). Thus, the EE of our SPH‐loaded beads could be improved by process optimization.

**TABLE 1:** Particle diameters and protein EEs of hydrogel beads obtained with different concentrations of alginate and chitosan.

Concentration of hydrogels		Bead characteristics	
Alginate (% w/v)	Chitosan (% w/v)	Diameter (mm)	EE (%)
2.0	0.00	1.01 ± 0.10^c^	15.28 ± 1.01^d^
2.0	0.25	1.26 ± 0.11^b^	43.88 ± 0.93^b^
2.0	0.50	1.24 ± 0.12^b^	45.07 ± 2.27^b^
2.0	0.75	1.25 ± 0.12^b^	45.28 ± 3.40^b^
3.0	0.00	1.03 ± 0.11^c^	27.17 ± 1.80^c^
3.0	0.25	1.53 ± 0.14^a^	61.00 ± 1.95^a^
3.0	0.50	1.52 ± 0.11^a^	50.82 ± 0.71^b^
3.0	0.75	1.53 ± 0.13^a^	47.80 ± 0.63^b^

*Note:* Data are shown as the mean ± SD of the experiments that were conducted in triplicate. Different superscripts within the same column indicate significant differences at *p* < 0.05 analyzed using an ANOVA and Duncan's multiple range test.

### 3.2 In Vitro Protein Release Behavior of SPH‐Loaded Beads

**FIGURE 1:**
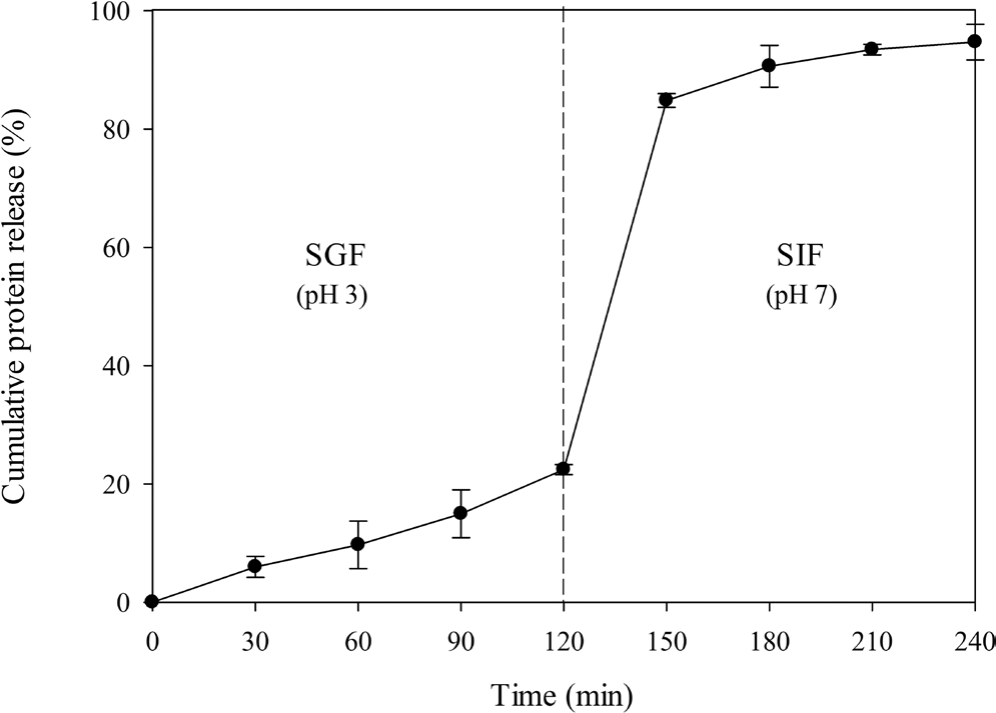
Protein release profile of SPH‐loaded alginate/chitosan composite beads during in vitro gastrointestinal digestion. Data are shown as the mean ± SD of the experiments that were conducted in triplicate.

The protein release profile of the SPH‐loaded beads during in vitro gastrointestinal digestion is shown in Figure 1. In the simulated gastric environment, the amount of protein liberated from these beads into the SGF was low (22.45% cumulative protein release after 2 h). The slow protein release from the beads observed in the SGF was attributed to the low swelling capacity of alginate hydrogel at acidic pH levels, making it difficult for protein to diffuse through the dense, thick matrix of the beads (Balanč et al. 2016). The formation of ionic bonds between alginate and chitosan created a strong physical barrier, which contributed to the high resistance of the beads to the SGF (Thongcumsuk et al. 2023). As expected, the amount of protein released increased when the beads were exposed to the SIF, with a burst protein release (~62% of total protein encapsulated) observed within the first 30 min of exposure. After this stage, the remaining protein (~10% of total protein encapsulated) was gradually liberated from the beads into the SIF, with a final accumulative protein release of 94.72%. This finding concurred with previous studies suggesting that extensive release of protein from alginate/chitosan composite beads took place in the SIF, resulting from the physiological pH levels of the small intestine (pH 6.0–7.5). The interchange of calcium ions stabilized the polymeric matrix of the beads, with cations present in the SIF (e.g., K^+^ and Na^+^) leading to the disintegration of the bead structure (Ling et al. 2019; Wang et al. 2019). The rapid protein release from the beads occurred because our core material as reconstituted SPH exhibited negative zeta potential over a wide range of pH, and was highly soluble in aqueous media (Balanč et al. 2016).

Alginate/chitosan composite beads can preserve the functionality of therapeutic protein substances (e.g., IgA and superoxide dismutase) and control their release in the gastrointestinal tract (Ren et al. 2017; Zhu et al. 2023). However, scant research has focused on the application of this encapsulation system for the protection of bioactive protein hydrolysates and/or peptides. To exert health benefits, bioactive peptides need to survive adverse environmental conditions and cross the intestinal epithelial barrier to reach the target organs in an intact and functional form (Aguilar‐Toalá et al. 2022). To primarily assess the functional protection conferred by the alginate/chitosan matrix, the antioxidant activities of both free and encapsulated SPH were compared following in vitro gastrointestinal digestion. We found that SPH retained its antioxidant potential effectively when encapsulated within hydrogel beads (Table S2). Post‐digestion retention rates for ORAC, FRAP, and DPPH radical scavenging activities exceeded 90%, indicating minimal degradation of peptide structure or conformation. In contrast, free SPH exhibited a significant decline in antioxidant activities, with reductions of up to 3.0‐fold compared to the original, undigested free SPH sample. Hence, this study supported the potential use of composite alginate/chitosan hydrogels for improving the bioavailability of bioactive peptides by efficient targeted delivery to the small intestine as the major absorption site. Nevertheless, the current findings are based on in vitro experiments, which represent a key limitation of the study. Therefore, further in vivo investigations are warranted to validate these results and confirm their physiological relevance.

### 3.3 Swelling Property of SPH‐Loaded Beads

**FIGURE 2:**
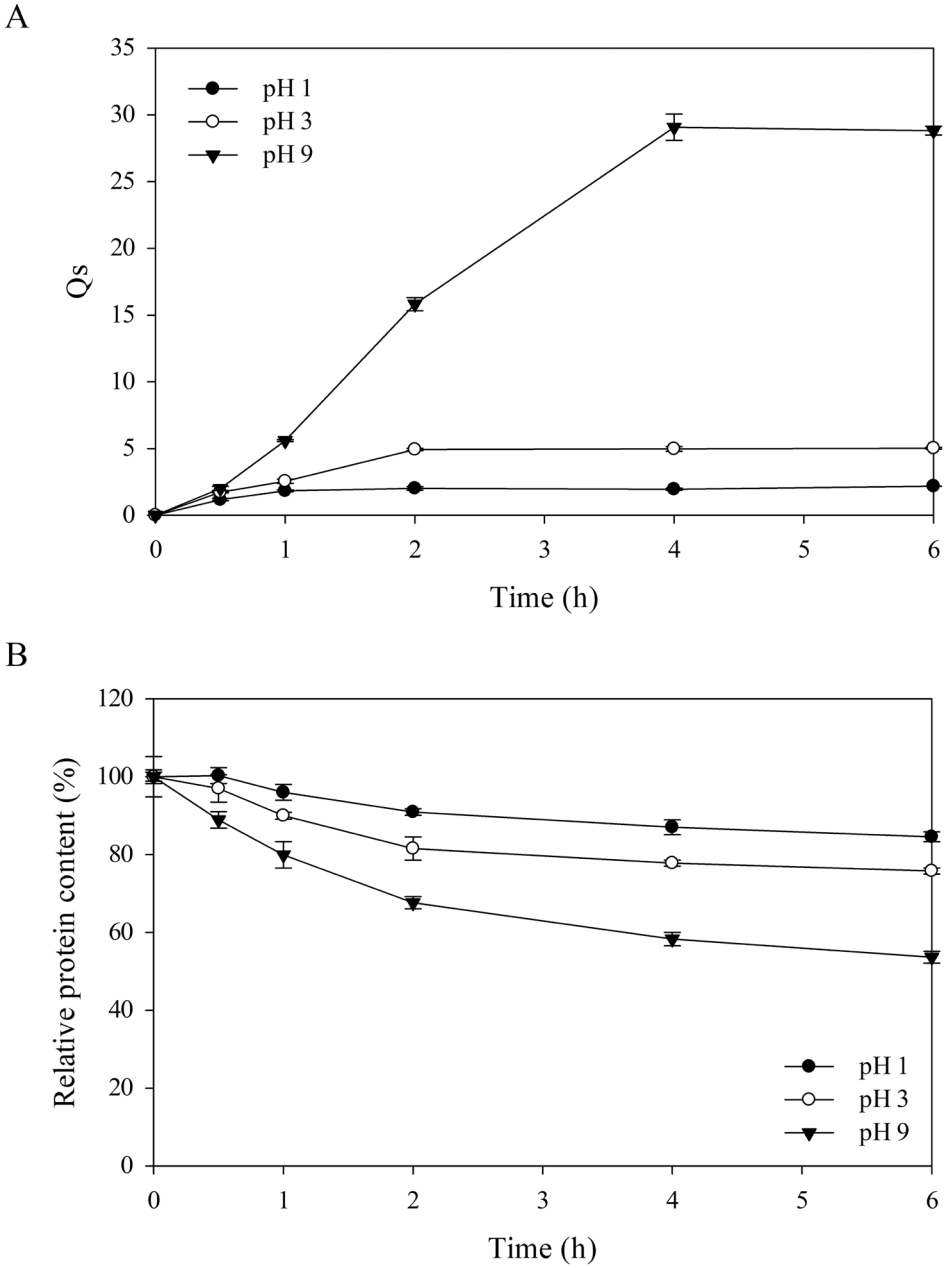
Changes in (A) the *Qs* value and (B) the relative protein content of SPH‐loaded alginate/chitosan composite beads following air‐drying process during swelling at different pH levels. Data are shown as the mean ± SD of the experiments that were conducted in triplicate. Data collected at pH 7.0 were not applicable due to the beads bursting.

Hydrogel beads prepared by ionotropic gelation are susceptible to physicochemical changes and microbial spoilage due to their high moisture content. Hence, a drying process was employed in this study to mitigate these risks and to enhance the stability and shelf‐life of our beads, particularly for purposes related to storage, transport, and packaging. Air‐drying at 50°C overnight reduced the moisture content of the SPH‐loaded beads from 94.78% ± 0.23% to 3.74% ± 0.15%, which was sufficient to ensure the microbiological safety of the food products (Afolabi 2014). The swelling property of dried SPH‐loaded beads was investigated at different pH levels. Changes in the *Qs* value and relative protein content of the dried beads after immersion in pH‐adjusted PBS are shown in Figure 2.

The dried beads had an average diameter of 0.54 ± 0.08 mm and exhibited a pH‐dependent swelling property (Figure 2A). The beads had low swelling capacity and reached equilibrium swelling within 2 h at pH 1.0 and 3.0 with maximum Qs values of 2.18 and 5.03, respectively. By contrast, the *Qs* value of the beads at pH 9.0 increased over time and reached the highest value of 29.07 at 4 h. Swelling of the beads under acidic conditions was limited compared with the swelling of the beads in an alkaline environment. At acidic pH, alginate molecules are partially protonated and form cross‐linked aggregates through intermolecular hydrogen bonding. This strong polymeric structure also forms a polyelectrolyte complex with chitosan, resulting in rigid polymer networks with low water absorption capacity (Wong et al. 2021). At alkaline pH, chitosan becomes uncharged, thus weakening the intermolecular forces between the polymer chains and facilitating the penetration of water into the hydrogel matrix (Ma et al. 2022). The dissociation of carboxylic groups of alginate in a basic solution also promotes the expansion of the polymer composite and attracts polar water molecules (Mohamed et al. 2017). Interestingly, the cross‐linked polymeric structure of the beads disintegrated rapidly at pH 7.0, with the loss of their spherical shape. The carboxylic groups of alginate were ionized at this pH level, leading to an increase in electrostatic repulsion between the polymers. This phenomenon resulted in the degradation of the hydrogel matrix and caused the burst protein release (Zhu et al. 2023). The pH‐dependent swelling characteristics of dried SPH‐loaded beads were in agreement with the behavior of freshly prepared bead samples observed under simulated gastrointestinal conditions (Figure 1), thus supporting the effective use of these dried beads for targeted protein delivery purposes.

The high relative protein contents (> 80%) detected during the first hour of swelling at pH 1.0, 3.0, and 9.0 indicated that our dried SPH‐loaded beads had good protein‐retention capacity (Figure 2B). Protein retention in the beads was highest at pH 1.0, followed by pH 3.0 and pH 9.0. Notably, the beads burst at pH 7.0, leading to a rapid release of protein. These results confirmed that the alginate‐chitosan matrix had greater rigidity in acidic environments compared to alkaline and neutral conditions, thereby reducing protein migration from the beads into the surrounding solution. At pH 3.0, the beads exhibited a comparable retention rate of protein to freshly prepared beads incubated in SGF, as presented in Figure 1. These results suggested the effectiveness of alginate/chitosan composite beads in retaining protein molecules at low pH levels, regardless of whether they were in a hydrated state or rehydrated form. After a swelling period of 6 h, beads rehydrated at pH 1.0 and 3.0 maintained their round shape and had high protein contents (~85% and ~76% of the initial protein content, respectively), while beads rehydrated at pH 9.0 possessed moderate protein content (~54%) compared to the initial protein value. The high structural stability of the beads at low pH levels facilitated the incorporation into acidic food products such as fruit juices, fermented drinks, and yogurts.

### 3.4 Surface Morphology of SPH‐Loaded Beads

**FIGURE 3:**
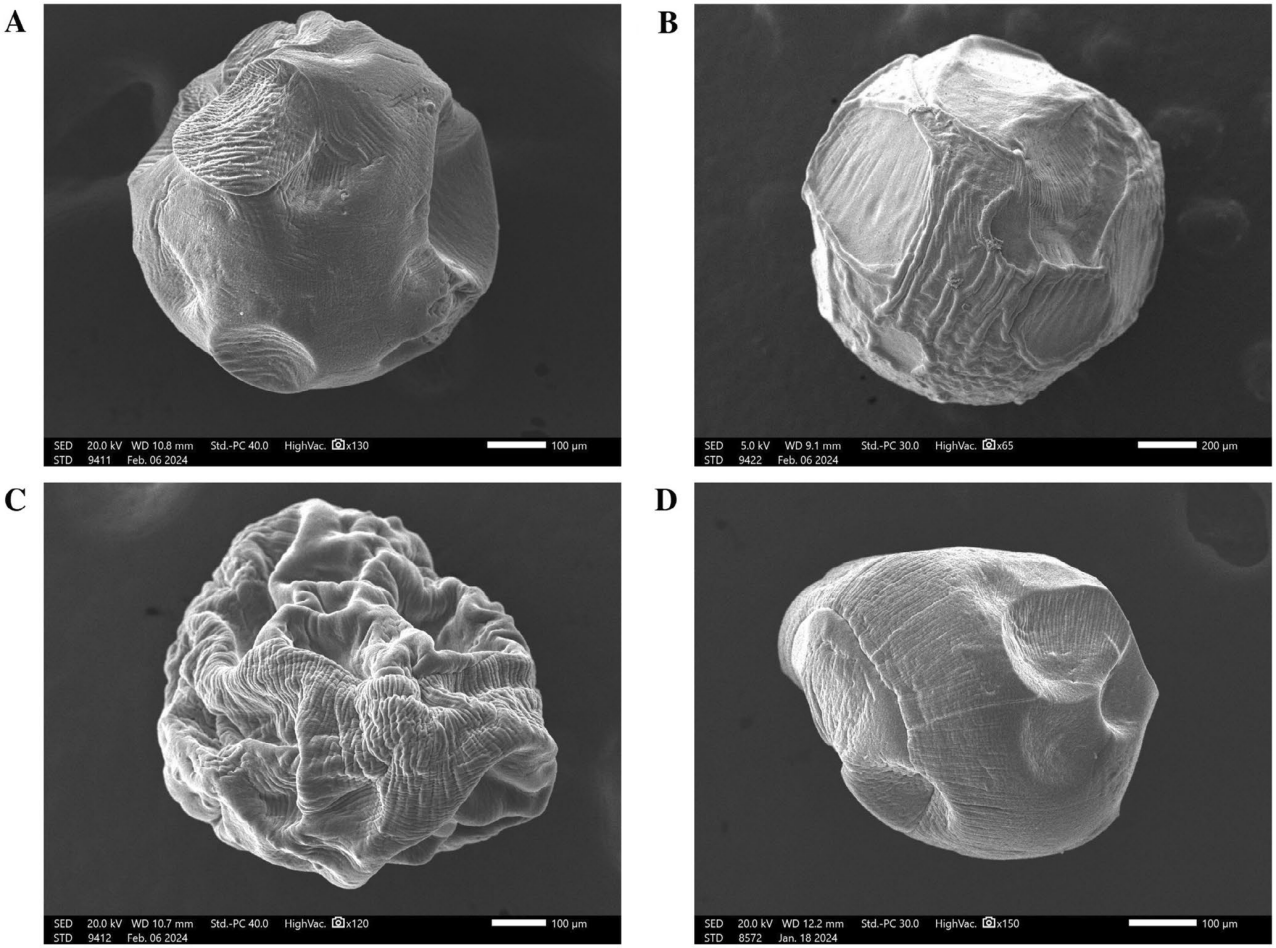
Scanning electron microscope images of alginate/chitosan composite beads: (A) empty bead without SPH, (B) SPH‐loaded bead, (C) air‐dried SPH‐loaded bead, and (D) air‐dried SPH‐loaded beads after rehydration at pH 3.0 for 2 h.

The scanning electron microscope images of alginate/chitosan composite beads prepared in this study are presented in Figure 3. Empty beads without SPH possessed a rough and highly porous surface (Figure 3A). Due to their low structural integrity, these beads were prone to deformation and collapse under vacuum conditions during scanning electron microscopy. In contrast, beads containing SPH exhibited a denser, more compact surface and maintained a spherical morphology (Figure 3B). This morphological difference indicated the significant contribution of the SPH to the structural rigidity and encapsulation performance of the beads. Interestingly, polymeric debris was observed on the surface of the SPH‐loaded beads, which may be attributed to the interaction of SPH with the alginate–chitosan side chains during the formation of the polymer blend matrix (Mohamed et al. 2017). The air‐drying process led to shrinkage of the polymeric network of the SPH‐loaded beads, resulting in amorphous beads with rugged surface features (Figure 3C). This phenomenon was also reported in previous studies involving alginate‐based systems (Azad et al. 2020; Balanč et al. 2016). Nevertheless, beads rehydrated at pH 3.0 for 2 h reverted to a nearly spherical shape, remained structurally intact, and were capable of entrapping SPH within their matrix (Figure 3D). This observation was consistent with the findings reported by Azad et al. (2020) and concurred with the aforementioned swelling property results, which showed that only a small amount of protein was released after 2 h of bead swelling at pH 3.0.

### 3.5 Bioactivities of Free and Encapsulated SPHs

In vitro antioxidant, anti‐hypertensive, and anti‐obesity activities of free and encapsulated SPHs were determined, with results shown in Table 2.

**TABLE 2:** Antioxidant and enzyme inhibitory activities of free and encapsulated SPHs.

SPH sample	Antioxidant activities (μmol TE/g_protein_)			Enzyme inhibition (%)	
	ORAC	FRAP	DPPH	ACE^1^	Lipase^2^
Free	1368.98 ± 48.21^a^	123.72 ± 3.42^a^	0.21 ± 0.02^a^	74.11 ± 3.42^a^	62.04 ± 2.28^a^
In fresh beads	1290.76 ± 66.23^ab^	122.11 ± 5.94^a^	0.20 ± 0.01^a^	71.07 ± 1.04^b^	62.77 ± 1.85^a^
In air‐dried beads	1251.60 ± 35.55^b^	118.19 ± 7.98^a^	0.21 ± 0.03^a^	70.96 ± 0.56^b^	60.63 ± 2.07^a^

*Note:* Data are shown as the mean ± SD of the experiments that were conducted at least in triplicate. Different superscripts within the same column indicate significant differences at *p* < 0.05 analyzed using an ANOVA and Duncan's multiple range test.

^1^ Protein concentration of all tested samples was 0.05 mg/mL.

^2^ Protein concentration of all tested samples was 2.00 mg/mL.

From the ORAC, FRAP, and DPPH radical scavenging assays, all SPH samples exhibited antioxidant activities at 1252–1369, 118–124, and 0.20–0.21 μmol TE/g_protein_, respectively, indicating their potency in reducing oxidation‐mediated stress within tissues and cells. Production and air‐drying of alginate/chitosan composite beads loaded with SPH under the studied conditions resulted in a decrease in ORAC activity of SPH, but these processes had no significant impact on FRAP and DPPH radical scavenging activities. The ORAC method based on fluorescence measurement offers greater sensitivity in assessing antioxidant potential than absorbance‐based antioxidant assays like FRAP and DPPH radical scavenging methods (Cao and Prior 1998). This was a plausible explanation for the significant reduction in ORAC values detected in SPH samples encapsulated in fresh and dried beads. The small reduction in antioxidant activities observed in the SPH samples that underwent the encapsulation process (< 6% reduction compared to free SPH) revealed that the gelation conditions used in this study were not detrimental to antioxidant compounds in the SPH. Similarly, previous studies successfully fabricated alginate‐based hydrogels to preserve antioxidant peptides with high antioxidant activity retention (Gallegos‐Tintoré et al. 2024; Kanbargi et al. 2017). The comparable antioxidant activities between SPH samples encapsulated in fresh and dried beads suggested that air‐drying at 50°C did not diminish the antioxidant potential of the encapsulated SPH, concurring with an earlier study reporting the insignificant effect of temperatures below 60°C on antioxidant peptides (Du et al. 2020). We found that empty alginate/chitosan composite beads exhibited mild antioxidant activities (~16%–20% of SPH's activities; data not shown), suggesting that the composite hydrogel used as a carrier material contributed to the enhanced antioxidant activities of the SPH‐loaded beads.

Both free and encapsulated SPHs showed inhibitory activities against ACE and lipase, the key enzymes relating to hypertension and obesity, respectively. Inhibition of ACE by the SPH samples was detected at a concentration of 0.05 mg_protein_/mL, at which the ACE inhibitory activity of free SPH (74.11% inhibition) was higher than the ACE inhibitory activities of its encapsulated counterparts (~71% inhibition). The reduced ACE inhibition observed in encapsulated SPH samples was attributed to partial amino acid degradation (e.g., glycine, proline, and valine) and/or conformational change of anti‐hypertensive peptides during the encapsulation process (Alvarado et al. 2019). By contrast, at a tested concentration of 2 mg_protein_/mL, no significant difference in lipase inhibition was observed between free (62.04% inhibition) and encapsulated SPH samples (~61%–63% inhibition). This discrepancy was probably due to differences in the amino acid sequences and the ability to interact with ionic molecules across peptide species (Jakubczyk et al. 2020). Insignificant changes in ACE and lipase inhibitory activities were found between SPHs encapsulated in fresh and dried alginate/chitosan composite beads, indicating that a drying temperature of 50°C did not cause any damage to ACE and lipase inhibitors present in the SPH. While peptide profiling was not conducted in this study, previous reports on the shrimp head composition of Pacific white shrimp identified aspartate, arginine, glutamate, and lysine as predominant amino acids (da Silva et al. 2017; Wu et al. 2021). These amino acids might contribute significantly to the bioactivities observed in the SPH, alongside other influencing factors such as peptide structure, molecular weight distribution, and the specific hydrolysis conditions employed (Kemsawasd et al. 2024). Unsurprisingly, empty alginate/chitosan composite beads exhibited only negligible levels of ACE and lipase inhibitions (~5% and ~9% of SPH activities, respectively; data not shown), and the carrier material did not contribute to the anti‐hypertensive and anti‐obesity effects of the SPH‐loaded beads.

The ionotropic gelation of alginate composites is a gentle process that does not alter the functional properties of sensitive molecules, making it an appropriate technique for encapsulating bioactive peptides and therapeutic proteins (Ling et al. 2019). Using alginate in combination with chitosan as a carrier material enhanced the stability of active core material, as a result of the formation of a strengthened physical barrier, known as the interphasic membrane, through ionic complexation of the two polymers (Ren et al. 2017). Our results also supported that the encapsulation of SPH in alginate/chitosan composite beads made by the ionotropic gelation process and air‐drying the beads with mild heat exposure did not have deleterious effects on the bioactivities of the encapsulated substance.

### 3.6 Storage Stability of Free and Encapsulated SPHs

**FIGURE 4:**
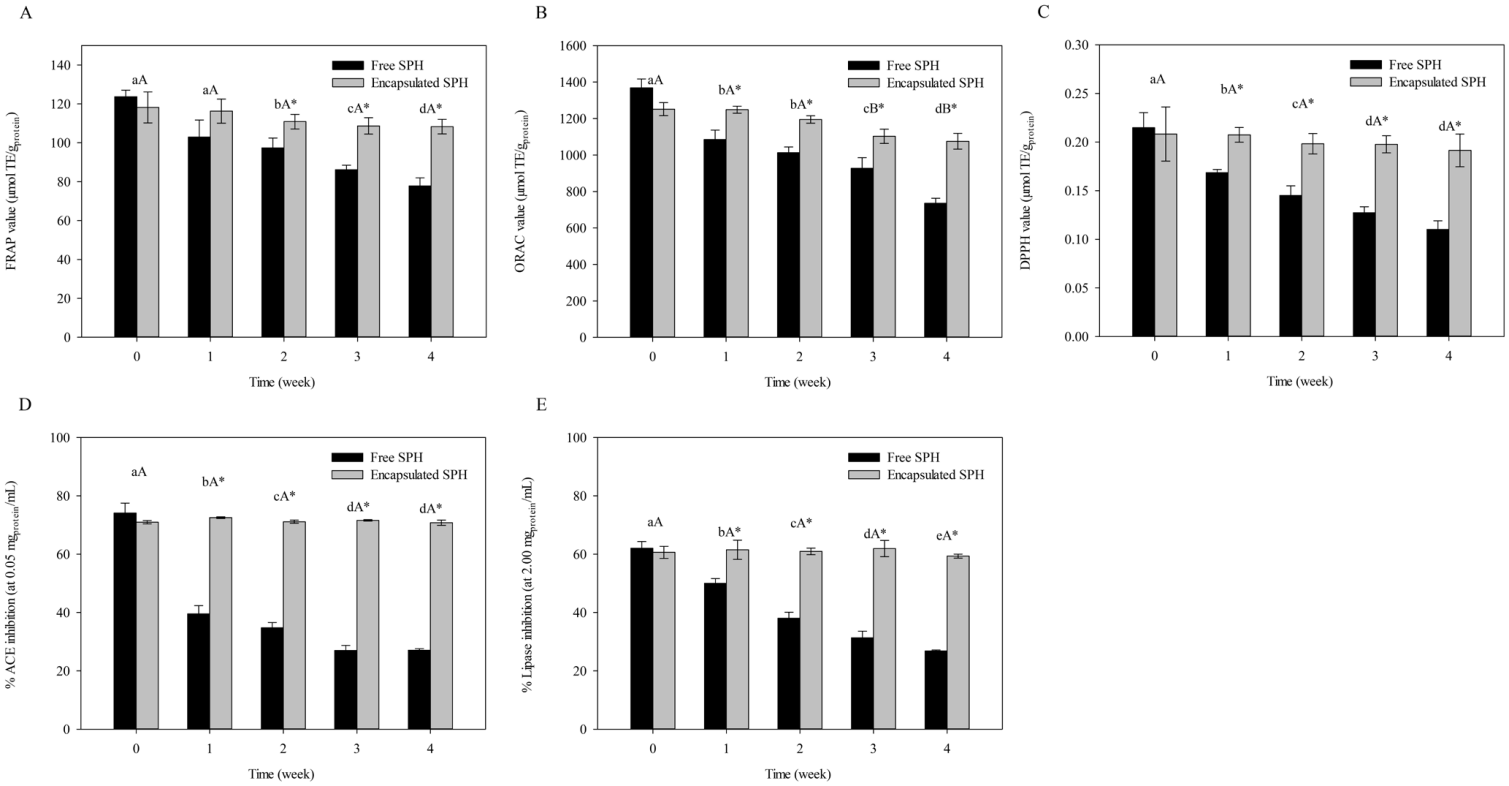
Changes in bioactivities of free and encapsulated SPHs during 4 weeks of refrigerated storage. The antioxidant activities investigated included (A) ORAC, (B) FRAP, and (C) DPPH radical scavenging activities. The enzyme inhibitory activities assessed included (D) ACE inhibition and (E) lipase inhibition. Data are shown as the mean ± SD of the experiments that were conducted in triplicate. For free and encapsulated SPH samples, different lowercase and uppercase letters above the bars denote significant differences between observation weeks (*p* < 0.05), respectively, as determined by ANOVA and Duncan's multiple range test. “*” indicates a significant difference between free and encapsulated SPHs within the same week (*p* < 0.05) analyzed using an unpaired *t*‐test.

The stability of free SPH and encapsulated SPH in dried beads during 4 weeks of refrigerated storage was assessed by monitoring changes in antioxidant and enzyme inhibitory activities. Results collected at 1‐week intervals are presented in Figure 4.

At the beginning of the storage period (week 0), there were no significant differences in antioxidant activities between free and encapsulated SPHs; however, as the storage time progressed (weeks 1–4), encapsulated SPH exhibited significantly higher ORAC, FRAP, and DPPH radical scavenging activities compared to free SPH (Figure 4A–C). Free SPH had low stability against oxidation and experienced significant decreases in ORAC, FRAP, and DPPH radical scavenging activities after 1 week of refrigerated storage amounting to 17%–22% of their initial values. The antioxidant activities of the non‐encapsulated form of SPH continued to decline during storage. After 4 weeks of refrigerated storage, the ORAC, FRAP, and DPPH radical scavenging activities of free SPH were retained at 46.27%, 37.13%, and 48.73%, respectively. Despite a significant decrease in the ORAC value after 3 weeks of refrigerated storage (~12% activity reduction), the FRAP and DPPH radical scavenging activities of the encapsulated SPH did not significantly change throughout 4 weeks of refrigeration. The retentions of antioxidant activities in encapsulated SPH were 85.94%, 91.63%, and 91.93% after the 4‐week storage period, as determined by the ORAC, FRAP, and DPPH radical scavenging assays, respectively. These results revealed that the encapsulation process, using a composite alginate/chitosan hydrogel as a carrier material, remarkably improved the oxidative stability of the SPH. Additionally, we monitored the protein content of the dried SPH‐loaded beads over the 4‐week refrigeration period and observed insignificant changes (data not shown), thereby confirming the absence of protein loss or premature release during storage. Our findings concurred with Kanbargi et al. (2017), who preserved antioxidant peptides during 30 days of refrigeration using alginate‐based hydrogels, with high retention of antioxidant activities (> 88%) detected at the end of the observation period. Xu et al. (2021) reported similar results, with the antioxidant properties of an encapsulated protein hydrolysate better maintained during storage compared to the free hydrolysate. These studies collectively demonstrated that the polymer coating used in the encapsulation acted as a barrier layer, shielding protein hydrolysates from oxidation.

Following refrigerated storage, the encapsulated SPH had significantly higher inhibitory activities against ACE and lipase compared to its free form at the same observation week (weeks 1–4), despite having comparable initial inhibitory activities (week 0) (Figure 4D,E). Significant decreases in the percentages of ACE and lipase inhibitions during refrigerated storage were observed in free SPH, with the retentions of inhibitory activities against ACE and lipase in free SPH after 4 weeks of refrigeration accounting for 36.53% and 43.25% of their initial inhibition values, respectively. On the contrary, ACE and lipase inhibitory activities of encapsulated SPH remained unchanged throughout the storage period (< 3% activity reduction). These results indicated that the alginate/chitosan composite beads produced in this study effectively minimized the degradation of ACE and lipase inhibitors in SPH during refrigerated storage. The enzyme inhibition capacity of protein hydrolysates is closely linked to their peptide compositions and influenced by various factors, such as molecular weight, hydrophobicity, charge, and amino acid sequences of the peptides (Mirzaei et al. 2020; Pei et al. 2022). Peptide aggregation and structural modifications may lead to conformational changes in peptide chains, profoundly impacting their interactions with enzymes (Mirzaei et al. 2020). Ensuring high affinity between the core and carrier materials is crucial for the formation of stable encapsulated peptide products that are capable of withstanding unfavorable conditions during processing and storage with limited diffusion losses (Mohan et al. 2015). The encapsulation of sensitive compounds like peptides within suitable matrices protects them from detrimental factors, such as oxygen, light, moisture, and temperature variations, that lead to storage degradation (Kanbargi et al. 2017; Xu et al. 2021). Many attempts have been made to utilize encapsulation techniques to preserve the enzyme inhibitory activities of protein hydrolysates and peptides, but existing studies have primarily focused on the protective effects of encapsulation against food processing and delivery conditions (Pei et al. 2022). Our results supported the application of composite hydrogel encapsulation systems that can prolong the enzyme inhibition potential of the peptide products. Preserving bioactivities during storage is crucial to ensure the functional efficacy of bioactive peptides as functional food ingredients and to maximize their potential health benefits for consumers. The alginate/chitosan composite beads developed in this study offered a simple and cost‐effective encapsulation approach for enhancing the functional performance and shelf‐life of SPH, particularly for applications in functional food and pharmaceutical products.

## 4 Conclusions

SPH, a shrimp‐derived protein hydrolysate, was encapsulated within a composite alginate/chitosan polymer, resulting in hydrogel beads with microparticle size. The beads had moderate EE, which could be improved by optimizing the encapsulation process. The beads exhibited pH‐responsive behavior, displaying high stability at gastric pH levels and serving as a suitable carrier for targeted protein delivery to the intestine. Drying the beads using mild heat was a viable method for prolonging their shelf‐life without damaging the polymeric structure and the encapsulated SPH. The antioxidant, anti‐hypertensive, and anti‐obesity activities of the encapsulated SPH were better maintained during 4 weeks of refrigeration compared to its non‐encapsulated counterpart, thus highlighting the effectiveness of the encapsulation system in protecting SPH from detrimental factors and preserving its bioactivities during refrigerated storage. Our findings emphasized the potential of an ionotropic encapsulation technique employing an alginate/chitosan composite as a carrier material to enhance the bioavailability and storage stability of protein hydrolysates, making them promising and cost‐effective functional ingredient candidates for incorporation into food and pharmaceutical products. However, future research should focus on characterizing the bioactive peptide profiles of SPH and elucidating their interactions within encapsulation matrices. In vivo studies are also needed to validate the current findings and inform the rational design of targeted delivery systems for specific food‐related applications.

## Author Contributions

**Sampurna Rai:** data curation (equal), formal analysis (supporting), investigation (equal). **Kanyawee Whanmek:** data curation (equal), formal analysis (supporting), investigation (equal). **Ploypailin Akanitkul:** investigation (supporting). **Angkansiri Deeaum:** investigation (supporting). **Thunnalin Winuprasith:** methodology (equal). **Varongsiri Kemsawasd:** conceptualization (equal), data curation (supporting), formal analysis (equal), methodology (equal), visualization (lead), writing – review and editing (equal). **Uthaiwan Suttisansanee:** methodology (equal). **Chalat Santivarangkna:** methodology (equal). **Suwapat Kittibunchakul:** conceptualization (equal), formal analysis (equal), methodology (lead), project administration (lead), writing – original draft (lead), writing – review and editing (equal).

## Conflicts of Interest

The authors declare no conflicts of interest.

## Supporting information


Data S1.

## Data Availability

Data will be made available on request.
