# Supplementary material for: Fabrication of Alginate/Chitosan Composite Beads for Improved Stability and Delivery of a Bioactive Hydrolysate From Shrimp ( Litopenaeus vannamei ) Head
Source: Food Sci Nutr. 2025 Jun 17;13(6):e70443. doi: 10.1002/fsn3.70443 (PMC12172002; doi:10.1002/fsn3.70443)
Supplement: Supplementary file 1 — Data S1. [file FSN3-13-e70443-s001.docx]

**Supplementary Material**

**Fabrication of alginate**/**chitosan composite beads for improved stability and delivery of a bioactive hydrolysate from shrimp (*Litopenaeus vannamei*) head**

Sampurna Rai ^a†^, Kanyawee Whanmek ^b†^, Ploypailin Akanitkul ^b^, Angkansiri Deeaum ^b^, Thunnalin Winuprasith ^b^, Varongsiri Kemsawasd ^b^, Uthaiwan Suttisansanee ^b^, Chalat Santivarangkna ^b^, Suwapat Kittibunchakul ^b, *^

^1^ Master of Science Program in Food Science for Nutrition (International Program),

Institute of Nutrition, Mahidol University, Nakhon Pathom 73170, Thailand

^2^ Institute of Nutrition, Mahidol University, Nakhon Pathom 73170, Thailand

^†^ These authors contributed equally to this work and share first authorship.

* Corresponding author:

E-mail address: suwapat.kit@mahidol.ac.th (S. Kittibunchakul)

**Preparation of protein hydrolysate with bioactivities by fermentation of Pacific white shrimp’s head**

Following our previous research presented in Kemsawasd et al. (2024), the head portions of Pacific white shrimp (*Litopenaeus vannamei*) were sourced from a shrimp-processing plant in Samut Sakhon, Thailand. The samples were kept refrigerated during transportation to preserve their freshness. Upon arrival, they were dried at 65 °C overnight and subsequently ground into a fine powder. Shrimp head broth was prepared by blending powdered shrimp head with glucose solution (5% w/v) at a powder-to-solution ratio of 1:5 (w/v). The resulting broth was adjusted to pH 7.0 with 5 N NaOH, sterilized at 121 °C for 15 min, and used as a fermentation medium. A successive co-culture fermentation using *Bacillus amyloliquefaciens* TISTR-1880 and *Lactobacillus casei* TBRC-388 was performed. *Bacillus* culture (~9 log CFU/mL, 6% v/v) were transferred into fermentation flasks (250 mL) containing sterile shrimp head broth (100 mL) and cultivated at 37 °C, 150 rpm for 3 days. Sequentially, *Lactobacillus* culture (~9 log CFU/mL, 6% v/v) were aseptically transferred into the *Bacillus* fermentation content. The fermentation was continued for another 2 days, and then terminated by centrifugation (10,000×*g*, 15 min, 4 °C) and freeze-drying. The resulting shrimp head protein hydrolysate (SPH) was rich in proteins (~70 g/100 g dry weight) and exhibited significantly enhanced antioxidant potential (up to 2.3-fold) compared to its non-fermented control. The SPH also showed inhibitory activities against angiotensin-converting enzyme, α-amylase and lipase (IC_50_ <0.05, <0.50 and <2.00 mg_protein_/mL, respectively), indicating its improved anti-hypertension, anti-diabetes and anti-obesity effects, respectively.

**Table S1.** Proximate composition of SPH sample.

| Component | Nutritive value (per 100 g dry weight) |
| --- | --- |
| Energy (kcal) | 340.36 ± 5.13 |
| Protein (g) | 75.32 ± 1.03 |
| Fat (g) | ND |
| Carbohydrate (g) | 9.77 ± 0.26 |
| Ash (g) | 14.91 ± 0.26 |

Data are shown as the mean ± SD of triplicate experiments. ND: not detected.

**Figure S1.** Zeta (ζ) potential of SPH sample at different pH levels. The tested solution was prepared by dissolving lyophilized SPH in deionized water to obtain 1 mg of protein per mL. The pH was adjusted with 1 M HCl or NaOH.

**Table S2.** Antioxidant activities of free and encapsulated SPHs following in vitro gastrointestinal digestion.

| SPH sample | Antioxidant activities (µmol TE/g_protein_) | | | | | | |
| --- | --- | --- | --- | --- | --- | --- | --- |
|  | Pre-digestion | | |  | Post-digestion | | |
|  | ORAC | FRAP | DPPH |  | ORAC | FRAP | DPPH |
| Free | 1294.58 ± 35.62 ^aA^ | 120.72 ± 4.27 ^aA^ | 0.17 ± 0.03 ^aA^ |  | 804.55 ± 13.46 ^bB^ | 40.44 ± 2.01 ^bB^ | 0.06 ± 0.00 ^bB^ |
| Encapsulated | 1196.60 ± 21.24 ^bA^ | 117.84 ± 6.57 ^aA^ | 0.19 ± 0.02 ^aA^ |  | 1223.49 ± 59.75 ^aA^ | 120.16 ± 8.49 ^aA^ | 0.17 ± 0.02 ^aB^ |

Data are shown as the mean ± SD of the experiments that were conducted at least in triplicate. For each antioxidant activity assay, different lowercase letters within the same column and different uppercase letters within the same row indicate statistically significant differences (*p* < 0.05), as determined by unpaired *t*-tests. Both free and encapsulated SPH were evaluated for their antioxidant activities after undergoing in vitro digestion using the INFOGEST static gastrointestinal model (Brodkorb et al., 2019). For the analysis of encapsulated SPH, dried SPH-loaded beads were first rehydrated in phosphate buffer solution (pH 3.0) for 2 h prior to digestion. The simulated digestive fluid obtained from empty beads served as the control for the encapsulated SPH, while digestive fluid supplemented with deionized water in an amount equivalent to the added SPH was used as the control for free SPH.

**References**

Brodkorb, A., Egger, L., Alminger, M., Alvito, P., Assunção, R., Ballance, S., Bohn, T., Bourlieu-Lacanal, C., Boutrou, R., Carrière, F., Clemente, A., Corredig, M., Dupont, D., Dufour, C., Edwards, C., Golding, M., Karakaya, S., Kirkhus, B., Le Feunteun, S., . . . Recio, I. (2019). INFOGEST static in vitro simulation of gastrointestinal food digestion. *Nature Protocols*, *14*(4), 991-1014. <https://doi.org/10.1038/s41596-018-0119-1>

Kemsawasd, V., Karnpanit, W., Thangsiri, S., Wongputtisin, P., Kanpiengjai, A., Khanongnuch, C., Suttisansanee, U., Santivarangkna, C., & Kittibunchakul, S. (2024). Efficient recovery of functional biomolecules from shrimp *(Litopenaeus vannamei)* processing waste for food and health applications via a successive co-culture fermentation approach. Current Research in Food Science, 9, 100850. [https://doi.org/10.1016/j.crfs.2024.100850](https://doi.org/https://doi.org/10.1016/j.crfs.2024.100850)
